# Is there any association between undesired children and health status of under-five children? Analysis of a nationally representative sample from Bangladesh

**DOI:** 10.1186/s12887-022-03489-7

**Published:** 2022-07-25

**Authors:** Md. Zakiul Alam, Md. Syful Islam

**Affiliations:** 1 Department of Population Sciences, University of Dhaka, Dhaka, 1000 Bangladesh; 2 Department of Population Science, Jatiya Kabi Kazi Nazrul Islam University, Trishal, Mymensingh, 2220 Bangladesh

**Keywords:** Undesired children, Child health, Childhood morbidity, Childhood mortality, Childhood malnutrition, Bangladesh

## Abstract

**Background:**

Child health, especially childhood mortality, is one of the critical indicators of human development. No child mortality is desirable, but it is still high in Bangladesh. We aimed to assess the effect of the child's desired status on childhood morbidity and mortality in Bangladesh.

**Methods:**

We used the data from the nationally representative cross-sectional *Bangladesh Demographic and Health Survey (BDHS) 2017–18* and restricted the analyses to children born in the past five years preceding the survey. We estimated the undesired status (excess in boy, girl, both, and parity) by subtracting an ideal number of children from the total live birth. We measured childhood mortality (perinatal, early neonatal, neonatal, post-neonatal, infant, child, and under-five mortality), morbidity (fever, diarrhea, cough, and acute respiratory infectious-ARI), nutritional problems (stunting, wasting, underweight, and low birth weight), and treatments (postnatal care, treatment for fever, diarrhea/cough, and vitamin A supplementation). Finally, we utilized the chi-square test and multilevel mixed-effects logistic regression analyses.

**Results:**

The prevalence of undesired children was 19.2%, 21.5%, 3.7%, and 25.4% for boys, girls, both boys and girls, and parity, respectively. Age, education, residence, division, and wealth index were significantly associated with undesired children. The prevalence of under-five mortality was 3.3% among desired children, almost double (5.4%) among undesired children. The likelihood of under-five mortality was [adjusted odds ratio (aOR): 2.05, *p* ≤ 0.001] higher among undesired children. Despite lower under-five mortality among higher socioeconomic status, the relative contribution of undesired children to under-fiver mortality was substantial. The undesired girl children were associated with an increased likelihood of moderately wasting (aOR: 1.28, *p* = 0.072), severely underweight (aOR: 1.41, *p* = 0.066), and low birth weight (aOR: 1.50, *p* ≤ 0.05). Moreover, the undesired children were 19% (*p* ≤ 0.05) more likely to be infected with fever. The undesired children had lower treatment for diarrhea and fever/cough and were less likely to get vitamin A supplementation (aOR: 0.71, *p* ≤ 0.001).

**Conclusions:**

The share of childhood morbidity, mortality, and malnutrition were higher among undesired children. Every child should be wanted, and no unwanted pregnancies are desirable; thereby, the government should reemphasize the proper use of family planning methods to reduce child mortality and malnutrition.

**Supplementary Information:**

The online version contains supplementary material available at 10.1186/s12887-022-03489-7.

## Background

Along with Sub-Saharan African and South Asian countries, unwanted fertility is one of the highest in Bangladesh [1]. The total fertility rate in Bangladesh is 2.3, but the wanted fertility rate is only 1.6. Unwanted childbearing has been associated with increasing poor child health; thus, reducing unwanted childbearing has been an essential factor for improving child health and survival [2, 3]. Substantial progress has been observed in child and infant survival worldwide [4–6]. In 1990, 1 in every 11 children died before reaching their fifth birthday, which has declined to 1 in every 26 children in 2018. Despite this substantial progress, around 15,000 children die every day before reaching their fifth birthday [7]. More than half of the deaths occur from preventable causes, and 90% of all deaths take place in developing countries, including Bangladesh [5, 7]. Malnourished children have a higher risk of death from common childhood illnesses such as pneumonia (acute respiratory infectious), diarrhea, and malaria. Overall, nutrition-related factors (stunting, wasting, underweight) are responsible for almost 45% of deaths of under-five children [7]. However, childhood mortality and morbidity, including malnourishment, might be higher among undesired children.

Moreover, the United Nations recognizes the must need to end preventable infant and child deaths between 2016 and 2030 [8], and the third goal of Sustainable Development Goals (SDGs) focuses on ensuring healthy lives and promoting wellbeing [9]. As of SDGs, all countries should reduce neonatal mortality and under-five mortality to 12 or fewer and 25 or fewer per 1000 live births within 2030, respectively [5, 6, 9]. The under-five mortality rate of 118 countries had already been below the SDG target. Still, for the remaining countries, mainly from Sub-Saharan Africa and Central and Southern Asia, progress and promises will need to be accelerated to achieve the target [5, 7]. Therefore, reducing unwanted pregnancy and childbearing will help in reaching the goal.

Bangladesh, the most densely populated country in South Asia, has substantial under-five mortality [1, 5]. In many developing countries, including Bangladesh, the leading causes of child mortality are pneumonia, diarrhea, birth complications, malnutrition, malaria, and neonatal sepsis [5, 6, 10, 11]. More than half of the diseases are treatable through simple and affordable interventions [7]. However, the health situation for unwanted children is even worse [12]. If a woman delivers more than she desires, then the children (either gender) may face a lack of proper caring due to conscious or unconscious neglect to the child and thus reduce mothers' ability to cope with child's everyday needs. This reluctance towards unwanted children may start from pregnancy and lead to the child's adverse health impacts [12, 13]. Shaka et al. showed that children from unintended pregnancies were three times more likely to be stunted in Ethiopia. The share of childhood mortality from unintended pregnancies was also larger in sub-Saharan Africa [2].

However, a good number of studies have been conducted to find out the determinants and differentials of child mortality, morbidity, and nutritional status of children aged under five in developed and developing countries [14–30]. Most of the studies identified the mother's age, the sex of children, birth order, household size, wealth status, education of parents, nutritional status of mothers, maternal care utilization, region, religion, residence as significant predictors of child health. However, few studies were on the relationship between undesired children and child health in developing countries, especially Bangladesh [2, 3, 31]. Every child should be wanted, [32], and wanted fertility will reduce child mortality and stabilize population growth. Moreover, women with unintended pregnancies were at higher risk of developing morbidity, including high blood pressure and anemia during pregnancy [33]. As a result, higher child and maternal mortality may occur. For instance, care-seeking behavior for mobility might be different for undesired children.

Bangladesh is one of the countries with the highest child marriage rate [1]. Due to child marriage, early childbearing contributes to having a higher number of unwanted children [34], which may increase the likelihood of poor maternal and child health outcomes [33]. The country also needs to achieve the third SDG of the ambitious child survival goal by 2030, which will be challenging with many undesired children. Thus, we first aimed to measure the prevalence of undesired children and their differentials. Then, we assessed the effect of undesired children on under-five child survival (neonatal, infant, and child mortality), morbidity and its treatment (acute respiratory infection, fever, diarrhea), postnatal care, and nutritional status (stunting, wasting, underweight, and overweight) by adjusting key socioeconomic covariates.

## Methods

### Source of data and inclusion criteria

We utilized the latest *Bangladesh Demographic and Health Survey (BDHS), 2017–18* [1]. The 2017–18 BDHS is the eighth of its type undertaken in Bangladesh as a part of an international program of measures DHS which follows two-stage stratified sampling with a response rate of 96.5%. The sample for the BDHS 2017–18 is nationally representative, and the detailed methodology will be found in the methodology section of the final report [1]. The 2017–18 BDHS collected information of 20,127 ever-married women, and all ever-married women were asked to provide detailed information about their desire and actual births in the past five years preceding the survey. We excluded the women who desired more than six children, as higher than six children are equivalent to natural fertility, and the number of children' up to god'. After the restriction, there were 7004 live births (numbers of children the woman had when the child died) of 6364 women five years preceding the survey. For the sensitivity analysis of the measures of undesired children, we also used whether the last child was wanted or not (*n* = 5668); the findings were available in the supplementary tables.

### Outcome variables

Outcome variables of this study were childhood mortality (including early neonatal, perinatal, neonatal, post-neonatal, infant, child, and under-five mortality), morbidity (including fever, diarrhea, cough, short and/or rapid breath, and ARI), treatments for common morbidities (fever and diarrhea), receiving PNC within two days after the birth, and malnutrition.

Childhood mortality was defined based on the timing of mortality in the last five years preceding the survey [35]. Stillbirths (SB) are pregnancies that last seven or more months in the womb and terminate in fetal death. Early neonatal mortality rate (ENMR) is the percentage of children who died at age 0–6 days after birth. Perinatal mortality is the sum of SB and ENMR. Neonatal mortality rate (NMR) is the percentage of death of children aged 0 to 28 days, while the post-neonatal mortality rate (PNMR) is the death of children aged between 29 and 364 days. NMR and PNMR together measure infant mortality rate (IMR). The under-five mortality rate (U5MR) is the death of children before reaching five years old.

All the morbidity information was collected having a presence of disease (yes/no) in the last two weeks preceding the survey. We measured the fever as the percentage of children under age five with the fever at any time within the previous two weeks preceding the survey. Cough and short and/or rapid breathing are also measured with the same procedures as fever. The acute respiratory infection (ARI) was calculated as the percentage of children under age five with chest-related short or rapid breathing symptoms and/or difficult breathing [35]. Along with the most common morbidities, we also considered the baby's postnatal checkup within two days after birth, treatment for diarrhea, treatment for fever/cough, and vitamin A.

DHS measures nutritional status based on both mother's estimate and anthropometric measurement. The child's size at the time of birth was measured according to reported birth weight, from either a written record or the mother's report. Low birth weight (LBW) is the reported birth weight below 2.5 kg regardless of gestational age. Using anthropometric measurement, we estimated stunting (height-for-age), wasting (weight for height), and underweight (weight for age) [35, 36].

### Predictor

In this study, we aimed to measure the effect of child's desired status of mother on childhood morbidity and mortality with socioeconomic differentials. Undesired children might directly be associated with parity, mother's age, education, and income, and we strived to consider it during measurement. For instance, the key predictor of this study was the 'desired status' categorized as excess in children (including excess in boys, excess in girls, excess in both, and excess in parity) and no excess in children. In DHS, women aged 15–49 were asked about the ideal number of children, including gender composition, whether they would like to have boys (number of boys), girls (number of girls), or either boys or girls (number of either gender) [1, 35]. We measured four types of undesired children, (a) being excess in boy child, (b) being excess in the girl child, (c) being excess in both boys and girls (dual excess), and (d) being excess in parity (excess in the total number of children). We measured undesired children using the conventional approach found in existing literature [2, 37–40]. The total number of live births (*LC*_*i*_) is the combination of boy (*B*_*i*_) and girl child (*G*_*i*_) of a mother provided in Eq. 1:

1 $${LC}_{i}={B}_{i}+{G}_{i}$$

The ideal number of children (*IC*_*i*_) is the combination of a number of a boy (*IB*_*i*_), girl (*IG*_*i*_), and either gender (*NG*_*i*_) (mother might prefer either gender of child) in Eq. 2:

2 $${IC}_{i}={IB}_{i}+{IG}_{i}+{NG}_{i}$$

If the total number of children exceeds the preferred number, then it would be excess in parity (*EP*_*i*_) for each birth five years preceding the survery in Eq. 3:

3 $${EP}_{i}={LC}_{i}-{IC}_{i}>0$$

If the number of boys exceeds the number of ideal boys plus the number of either sex, it would be excess in the boy (*EB*_*i*_) provided in Eq. 4. A similar approach would be for excess in girls (*EG*_*i*_) provided in Eq. 5. Finally, if it exceeds both the number of boys and girls, it would be dual excess (*DE*_*i*_) provided in Eq. 6.

4 $${EB}_{i}={B}_{i}-{(IB}_{i}+{NG}_{i})>0$$

5 $${EG}_{i}={G}_{i}-{(IG}_{i}+{NG}_{i})>0$$

6 $${DE}_{i}={EB}_{i} \& {EG}_{i}$$

Moreover, we also utilized the built-in BDHS variable "whether wanted last pregnancy" coded as "wanted then," "wanted later," and "wanted no more" for the sensitivity analysis of the measures of undesired children, which suffer more from rationalization (as the unwanted child may be wanted after birth).

### Other covariates

We selected other covariates based on the existing literature [14–17, 41] and demographics, socioeconomic, spatial, and programmatic factors affecting the health status of children. Demographic and health factors were also considered, including the current age of the mother, age of child/ year of birth, parity/ birth order, and sex of the child. We categorized the current age of women and age of motherhood into < 20, 20–34, and 35–49 years which was also used in other studies [14, 15, 41].

We included women's and husbands' education (categorized as no education, primary, secondary, and higher), wealth index, employment status, women empowerment, and religion as socioeconomic factors. The wealth index, which is used to assess the household's socioeconomic status, was constructed from data of household possessions using the principal component analysis and divided into three groups (poor, middle, and rich) based on overall asset ownership. We measured women's participation in household decision making using four pieces of information: the person who usually decides on the respondent's health care, the person who usually determines on large household purchases, the person who usually decides on visits to family or relatives, and the person who usually decides what to do with money husband earns. The Cronbach's alpha value was 0.810, suggesting very good internal consistency. We categorized participation as high if all the decision was made by the respondent alone, moderate empowerment if all the decision was made jointly with the husband or others, and low empowerment if husbands or others made the decision. Attitudes toward wife-beating were also measured using five items. A husband is justified in hitting or beating his wife if she: burns the food, argues with him, goes out without telling him, neglects the children, and refuses to have sexual intercourse with him (The Cronbach's alpha value was 0.673). Religion was categorized as Muslim and non-Muslim (Hindu, Buddhist, or Christian) due to most Muslims in Bangladesh.

Administrative division (eight divisions: Barisal, Chattogram, Dhaka, Khulna, Rajshahi, Rangpur, and Sylhet) and place of residence (whether residing in the urban or rural area) are two major spatial factors we included in the study. Moreover, we had antenatal care (ANC) and postnatal checkup (PNC) (also used as a dependent variable for baby) from medically trained providers, home visits by family planning (FP) workers in the last six months, and access to any media (women's access to any of these: TV, Radio, Newspaper or Magazines) for FP (family planning) information as programmatic factors affecting child health in Bangladesh. As DHS collected the information for the last birth, ANC and PNC were categorized as 'yes,' 'no,' and 'unknown,' which was also used by another researcher [15]. We also used community-level education and wealth as the mean of individual level.

### Statistical analysis

We performed both bivariate and multiple variables analyses. For bivariate analysis, we used the chi-square test. Since DHS data has a hierarchical structure with different levels: individuals nested within communities, and individuals within a cluster might be more similar than individuals in the rest of the country. It implies that we need to consider variability between clusters. Based on the cut-off probability value of ≤ 0.25 of the simple logistic regression, we used multilevel (mixed-effects) multiple logistic regression analyses to identify the effect of undesired children on childhood morbidity and mortality in Bangladesh. We run five models [Empty/null, Individual-level (child level), Mother and family level, Community-level (cluster level) model, and Final model)] to estimate the child, mother, household, and community-level factors and random intercept of between-cluster variation.

The Empty model was run to test the random effect of between-cluster variability without any independent variable. The intraclass correlation coefficient (ICC) was derived from the between-cluster and within-cluster variability. The Individual-level factors model examined the effects of child-level characteristics on child health outcomes. Besides, the ICC was observed if there was a decline in the between-cluster variability after adding individual factors to the empty model. The Mother and Family level model hold the characteristics of mother and household, whereas the Community-level factors model contains only characteristics of clusters, not individuals, mother, or household. Finally, all the child, mother, household, and clusters characteristics were concurrently fitted to one model to reveal their net fixed and random effects. The 'melogit' code was employed for analysis using KR (Children's Recode) file for multilevel analysis in Stata 16.

We used the variance inflator factor (VIF) to examine the instability of the effect size of predictors as the result of multicollinearity among the independent variables. The educational attainment of the husband, visits by FP workers, ANC, and PNC were excluded from the multiple analyses. The fixed-effects estimated the association between the child health outcomes and undesired status presented as Adjusted Odds Ratio (aOR) with their 95% confidence intervals (C.I.s). The random effects were reported as the intra-community correlation coefficient (ICC), the percentage variance explained by the higher level (community-level variables). The proportional change in community variance (PCV) expresses the change in the community level variance between the empty model (Model 1) and the consecutive models.

## Results

### Undesired children in Bangladesh

**Fig. 1:**
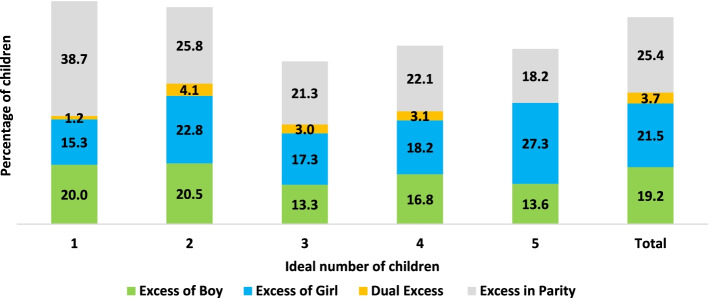
Percentage of undesired children by types of undesirability

About 37% of women had a lower distance to the ideal family size, while about 27% had a higher distance. The rest of the women had equal distance (zero distance) to the ideal family size. Lower and equal distances expressed the desired number of children, while the higher distance showed the undesired number of children. The overall undesired children (excess from ideal number) were 19.2%, 21.5%, 3.7%, and 25.4% for boys, girls, both boys and girls, and parity, respectively (Fig. 1). The excess in parity was the highest observed among single parity women, followed by parity two. The highest excess in boys was observed among two parity women, whereas the highest in girls was seen among the five. Similarly, the prevalence of mistimed or wanted children was 23.2% (wanted later- 14.8% and wanted no more- 8.4%) (see supplementary tables).

Table 1 provides the undesired children by selected background characteristics of the study population. Most of the socioeconomic characteristics were significantly associated with undesired children. The prevalence of undesired children was the highest among adult mothers (aged 35–49 years). The excess of boys, girls, both boys and girls, and parity were 43.2%, 55.8%, 22.6%, and 73.6% among the mothers aged 35–49. The women from urban areas had a higher prevalence of undesired children. The rate of undesired children was higher among Muslim women. It was pretty exciting but plausible that non-educated and poor women had a higher rate of undesired children than others. Working women had higher undesired children than non-working, but women having access to any media for FP had the lower one.

**Table 1:** Percentage of undesired children by selected socioeconomic characteristics

Background characteristics	Excess of boy	Excess of girl	Dual excess	Excess of parity	Number
**Sex of last child**	^***^	^***^			
Male	29.1	10.6	3.9	24.7	3694
Female	8.3	33.7	3.5	26.2	3309
**Year of birth of children**					
2014	21.7	22.3	4.3	26.6	1706
2015	18.6	19.6	3.6	25.1	1731
2016	17.5	22.7	3.2	24.9	1765
2017	19.0	21.3	3.5	24.9	1729
2018	25.0	26.4	12.5	31.9	72
**Current age of women**	***	***	***	***	
15–24	8.4	9.4	0.4	5.8	3537
20–34	28.2	31.0	5.5	41.4	3003
35–49	43.6	51.9	18.1	71.5	463
**Age of motherhood**	^***^	^***^	^***^	^***^	
11–19	4.7	4.4	0.0	2.1	1860
20–34	23.4	26.0	4.0	31.5	4851
35–49	43.2	55.8	22.6	73.6	292
**Place of residence**	^**^		^**^	^**^	
Urban	17.1	20.1	3.0	23.3	1916
Rural	20.0	22.1	4.0	26.2	5088
**Division (Region)**		^*^	^***^	^***^	
Barisal	20.1	19.3	2.6	28.3	378
Chattogram	19.2	22.3	4.3	27.1	1480
Dhaka	18.2	21.6	4.1	23.7	1812
Khulna	17.4	21.0	2.2	21.6	625
Rajshahi	22.7	22.5	5.7	29.4	599
Rangpur	18.2	17.5	2.0	22.1	817
Sylhet	19.5	23.6	2.7	27.5	730
**Religion**	^***^		^**^	^***^	
Muslim	19.7	21.8	3.9	26.1	6429
Other	13.6	18.6	1.4	17.3	574
**Household wealth index**	^***^	^***^	^***^	^***^	
Poor	22.5	24.7	5.1	31.2	2899
Middle	18.7	20.6	3.7	23.3	2743
Rich	13.4	16.5	0.9	17.3	1361
**Educational level of women**	^***^	^***^	^***^	^***^	
No education	35.1	39.0	13.6	51.5	478
Primary	25.1	27.2	6.1	35.6	1989
Secondary	16.7	20.0	2.0	21.1	3417
Higher	9.8	8.7	0.3	9.3	1120
**Husband's education level**	^***^	^***^	^***^	^***^	
No education	30.9	31.8	9.1	45.5	990
Primary	20.9	24.5	4.6	28.4	2363
Secondary	15.7	19.2	2.2	20.4	2293
Higher	13.3	12.6	0.7	13.2	1234
**Access to any media for FP**	^**^		^**^	^***^	
No	19.9	21.9	4.0	26.5	5828
Yes	16.0	19.7	2.5	20.2	1176
**Working status of women**	^***^	^***^	^**^	^***^	
No	16.5	19.3	3.1	21.5	4249
Yes	23.4	25.0	4.7	31.4	2754
**Women's participating in household decision making**	^**^				
Low	19.2	18.5	1.9	26.2	260
Moderate	19.9	22.5	3.9	26.3	5821
High	15.2	16.6	3.3	19.5	922
**Wife beating justified**				^**^	
No	19.1	21.6	3.9	24.8	5711
Yes	19.8	21.4	3.0	28.3	1292
**Total**	19.2	21.5	3.7	25.4	7003

***, ** and * denotes <0.001, <0.01, and <0.05 level of significance of chi-square tests

### Childhood mortality, morbidity, and nutritional problem by undesired status

#### Childhood mortality

Table 2 presents the childhood mortality rate by the desired status of children in Bangladesh. The prevalence of early neonatal mortality was 2.4%, with 2.0% among desired parity and 3.4% among excess in undesired parity. The perinatal mortality rate was 4.5%, with a variation of 4.4% among desired children and 4.9% among undesired children. However, neonatal mortality was 4.5% among excess in undesired boys but only 2.5% among desired boys. Between excess and non-excess in both children, the prevalence of infant mortality was 6.5% and 3.6%, respectively. Moreover, the prevalence of under-five mortality was 3.3% among desired children, almost double (5.4%) among undesired children.

**Table 2:** The prevalence (%) of morbidity and mortality among births in past five years by desired status of children in Bangladesh

Childhood Mortality and Morbidity	Excess in Boy		Excess in Girl		Dual Excess		Excess in Parity		Total	Number
	**No**	**Yes**	**No**	**Yes**	**No**	**Yes**	**No**	**Yes**		
**Indicators of Child Morbidity: Most Common Disease in Childhood**										
Diarrhea in last two weeks	5.6	5.3	5.7	4.7	5.6	3.7	5.6	5.3	5.5	6736
Fever in last two weeks	33.9	37.6	34.1	36.3	34.5	38.1	33.6	37.6	34.6	6737
Cough in last two weeks	36.6	38.8	37.5	35.4	37.0	37.3	37.0	37.2	37.0	6736
Short, rapid breaths	13.8	15.9	14.6	12.5	14.1	16.8	14.0	14.7	14.2	6736
Acute Respiratory Infection	3.1	3.7	3.3	2.8	3.2	3.3	3.0	3.7	3.2	6736
**Indicators of Child Morbidity: Childhood Nutritional Status**										
Severely Stunted	9.1	10.5	8.9	10.9	9.3	11.7	8.5	11.8	9.3	6279
Moderately Stunted	30.6	35.3	30.7	34.6	31.2	38.3	30.2	35.3	31.5	6280
Severely Underweight	4.1	4.6	3.9	5.2	4.0	8.7	3.8	5.2	4.2	6480
Moderately Underweight	20.0	25.1	20.3	23.6	20.6	31.9	19.8	24.5	21.0	6481
Severely Wasted	1.6	2.2	1.7	1.7	1.7	2.3	1.6	2.0	1.7	6263
Moderately Wasted	8.5	8.3	8.3	9.1	8.4	10.1	8.4	8.6	8.4	6263
Low Birth Weight (LBW)	15.7	14.5	14.5	20.9	15.3	29.3	14.7	19.5	15.5	2330
**Indicators of Child Mortality**										
Early Neonatal Mortality	2.0	3.9	2.4	2.4	2.3	5.0	2.0	3.4	2.4	7004
Perinatal Mortality	4.3	5.3	4.6	4.2	4.4	6.9	4.4	4.9	4.5	7003
Neonatal Mortality	2.5	4.5	2.8	2.9	2.7	5.4	2.5	3.9	2.8	7003
Post Neonatal Mortality	0.8	1.5	0.9	0.9	0.9	0.8	0.7	1.5	0.9	7003
Infant Mortality	3.2	5.9	3.7	3.8	3.6	6.5	3.2	5.4	3.7	7002
Child Mortality	0.1	0.0	0.1	0.1	0.1	0.0	0.1	0.1	0.1	7004
Under 5 Mortality	3.3	5.9	3.8	3.8	3.7	6.5	3.3	5.4	3.8	7004

#### Childhood nutritional problem

More than 31.0% of children under age five were stunted, 8.4% were wasted, and 21% were underweight (Table 2). The prevalence of severely stunting, wasting, and underweight was 9.3%, 1.7%, and 4.2%, respectively. Similarly, the prevalence of LBW was 15.5%. The prevalence of stunting, wasting, underweight, and LBW was higher among undesired children. On the other hand, the prevalence of overweight was higher among the desired children.

#### Childhood morbidity

We found that 5.5% and 34.6% of children had experienced diarrhea and fever in the last two weeks preceding the survey, respectively (Table 2). About 37% and 14.2% of children had a cough and short or rapid breath. The prevalence of symptoms of ARI was 3.2%. However, most childhood mortality and morbidly were also higher among undesired children.

#### Postnatal care, vaccination and treatment for most common childhood morbidities

**Fig. 2:**
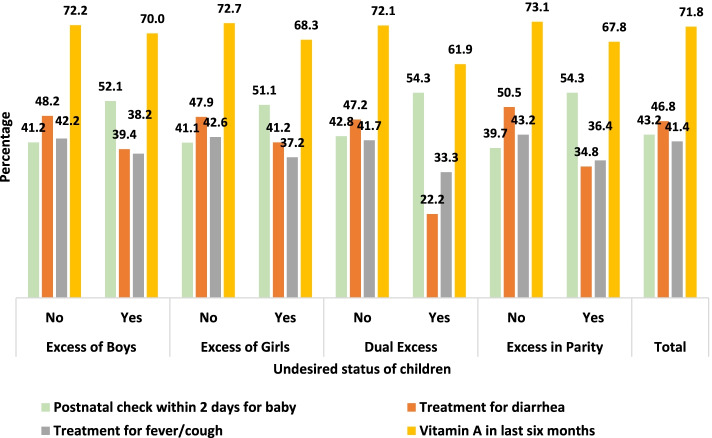
Seeking postnatal checkup for baby within 2 days and medical treatment for common illnesses by desired status of children in Bangladesh

Figure 2 illustrates postnatal checkups for babies within two days after birth and medical treatment for common illnesses by the desired status of children in Bangladesh. Forty-three percent of children received postnatal care within two days after birth, which was lower among desired children. However, there was a significant treatment variation for diarrhea and fever/cough by the desired status, where undesired children had lower treatments than others. In the context of vaccination, 71.8% received vitamin A within six months preceding the survey, which was lower for the undesired children.

### Morbidity, and nutritional problem

We carried out the generalized estimating equation analysis (multilevel mixed-effects logistic regression analyses) to assess the effect of undesired children on childhood mortality, morbidity, and nutritional problems. The results (provided in Table 3) showed a significant association between childhood mortality and undesired children ('no undesired children' was the reference category) in Bangladesh. The undesired boys were significantly associated with an increased likelihood of infant mortality (aOR: 2.03, 95% C.I.: 1.44–2.85, *p* ≤ 0.001) than desired boys. The probability of under-five mortality was two times higher (aOR: 2.05, 95% C.I: 1.43–2.95, *p* ≤ 0.001) among undesired children than that of the desired children.

**Table 3:** Generalized estimation of morbidity and mortality among births in past five years by desired status children in Bangladesh

Childhood Mortality and Morbidity	Excess in Boy	Excess in Girl	Excess in Parity
	**aOR (95% C.I.) **^**a**^	**aOR (95% C.I.)**^**a**^	**aOR (95% C.I.)**^**a**^
**Mortality**			
Early Neonatal Mortality	2.48 (1.63, 3.76) ^***^	1.37 (0.79, 2.38)	2.63 (1.64, 4.24) ^***^
Neonatal Mortality	2.05 (1.41, 2.99) ^***^	1.22 (0.76, 1.97)	2.10 (1.36, 2.26) ^***^
Post Neonatal Mortality	1.89 (0.90, 3.93)	0.80 (0.40, 1.60)	2.08 (1.09, 3.98) ^*^
Infant Mortality	2.03 (1.44, 2.85) ^***^	1.09 (0.73, 1.64)	2.12 (1.47, 3.04) ^***^
Under 5 Mortality	1.96 (1.39, 2.77) ^***^	1.08 (0.72, 1.60)	2.05 (1.43, 2.95) ^***^
**Nutrition **^**b**^			
Severely Stunted	0.92 (0.71, 1.18)	1.10 (0.87, 1.41)	1.17 (0.91, 1.51)
Moderately Stunted	1.05 (0.89, 1.24)	1.05 (0.89, 1.24)	1.02 (0.87, 1.19)
Severely Underweight	1.07 (0.72, 1.59)	1.41 (0.98, 2.05)	1.35 (0.95, 1.91)
Moderately Underweight	1.14 (0.94, 1.36)	1.11 (0.89, 1.28)	1.06 (0.88, 1.28)
Severely Wasted	1.49 (0.88, 2.51)	1.16 (0.67, 2.01)	1.35 (0.77, 2.37)
Moderately Wasted	0.86 (0.66, 1.14)	1.28 (0.98, 1.67)	1.01 (0.77, 1.34)
Low Birth Weight (LBW)	1.04 (0.71, 1.53)	1.50 (1.04, 2.18)^*^	1.47 (1.02, 2.11) ^*^
**Morbidity **^**c**^** and treatment**			
Diarrhea in last two week	0.88 (0.62, 1.25)	0.86 (0.63, 1.17)	1.00 (0.74, 1.36)
Fever in last two weeks	1.16 (0.99, 1.36)	1.12 (0.94, 1.33)	1.19 (1.02, 1.40) ^*^
Acute Respiratory Infection	1.04 (0.67, 1.63)	0.91 (0.59, 1.41)	1.24 (0.82, 1.87)
Postnatal checkup of baby within 2 days	1.26 (1.03, 1.54) ^*^	1.17 (0.97, 1.40)	1.39 (1.14, 1.68) ^***^
Treatment for fever/cough	0.86 (0.66, 1.14)	1.05 (0.80, 1.39)	0.93 (0.72, 1.18)
Vitamin A in last six months	0.83 (0.68, 1.01)	0.81 (0.68, 0.99) ^*^	0.71 (0.60, 0.84) ^***^

***Note:*** *, **, and *** denotes ≤ *0.05,* ≤ *0.01, and* ≤ *0.001 level of significance; aOR denote adjusted odds ratio, C.I. means confidence interval. a: Adjusted analyses (or aOR) controlled for age of child, sex of child, age of motherhood, religion, respondent's education, household wealth, access to any media for FP, working status of women, women's participation in major decision making, and women's attitude toward wife beating as individual and household level variables; whereas, place of residence, division, community-level education and wealth as community-level variables. b: childhood diseases were also controlled for childhood malnutrition. c: childhood malnutrition was also controlled for childhood diseases*

The undesired girls were significantly associated with an increased likelihood of being severely underweight, moderately wasted, and LBW than desired girls. If the girl children were excess, they were 41% (95% C.I.: 0.98–2.05, *p* = 0.066), 28% (95% C.I.: 0.98–1.67, *p* = 0.072), and 50% (95% C.I.: 0.97–2.03, *p* = 0.029) more likely to be severely underweight, wasted, and LBW, respectively.

We also found that undesired children were 19% (95% C.I.: 1.02–1.40, *p* ≤ 0.05) more likely to be infected with fever. In contrast, undesired children were less likely to get vitamin A supplementation (aOR: 0.71, 95% C.I.: 0.60–0.84, *p* ≤ 0.001). However, the postnatal checkup for babies within two days after birth was higher among children who were excess in parity (aOR: 1.39, 95% C.I.: 1.14 to 1.68, *p* ≤ 0.001).

The model fits statistics of multilevel modeling are provided in Table 4. Results showed variation in the likelihood of child health outcomes by an individual across clusters. According to the Intra-community correlation coefficient (ICC), 7% of the total unexplained variance in the odds of under-five mortality. The ICC) of the final model (individual and community level factors) implied that 4% of the total unexplained variance in the under-five mortality could be attributed to community characteristics. Due to simultaneous effects of both individual and community-level factors, the PCV found 35.3% of the variance in the log-likelihoods of under-five mortality of boys (27.5% and 40.3% for girls and parity) across communities.

**Table 4:** Random effects of multilevel logistic regression analyses

Indicators	Random effects						
	**ICC: Empty model (RC)**	**Excess in Boy**		**Excess in Girl**		**Excess in Parity**	
**Mortality**		**ICC: Final model**	**PCV (%)**	**ICC: Final model**	**PCV (%)**	**ICC: Final model**	**PCV (%)**
Early Neonatal Mortality	0.14	0.11	24.7	0.12	21.4	0.11	28.9
Neonatal Mortality	0.11	0.08	26.4	0.09	20.4	0.08	26.2
Post Neonatal Mortality	0.22	0.16	32.4	0.16	28.7	0.14	39.8
Infant Mortality	0.06	0.03	46.2	0.04	35.6	0.03	51.7
Under 5 Mortality	0.07	0.04	35.3	0.05	27.5	0.04	40.3
**Morbidity and treatment**							
Diarrhea in last two weeks	0.06	0.04	36.8	0.04	36.6	0.04	37.3
Fever in last two weeks	0.06	0.05	13.4	0.05	13.2	0.05	13.4
Acute Respiratory Infection	0.12	0.08	34.4	0.08	34.0	0.08	35.0
Postnatal checkup of baby	0.26	0.12	62.2	0.12	62.3	0.12	60.6
Treatment for fever/cough	0.11	0.08	24.7	0.08	26.9	0.08	25.2
Vitamin A in last six months	0.10	0.11	-3.8	0.11	-4.5	0.11	-5.3
**Nutrition**							
Severely Stunted	0.10	0.06	48.4	0.06	47.9	0.06	47.1
Moderately Stunted	0.05	0.03	51.6	0.03	51.8	0.03	51.6
Severely Underweight	0.10	0.06	39.0	0.07	38.6	0.07	38.4
Moderately Underweight	0.07	0.04	46.3	0.04	46.2	0.04	46.0
Severely Wasted	0.15	0.08	47.2	0.08	48.4	0.09	45.6
Moderately Wasted	0.09	0.08	10.1	0.08	10.4	0.08	11.0
LBW	0.08	0.06	23.2	0.06	23.8	0.06	21.6

*RC* Reference category, *ICC* Intra-class/community correlation coefficient, *PCV* Proportional change in community variance

**Fig. 3:**
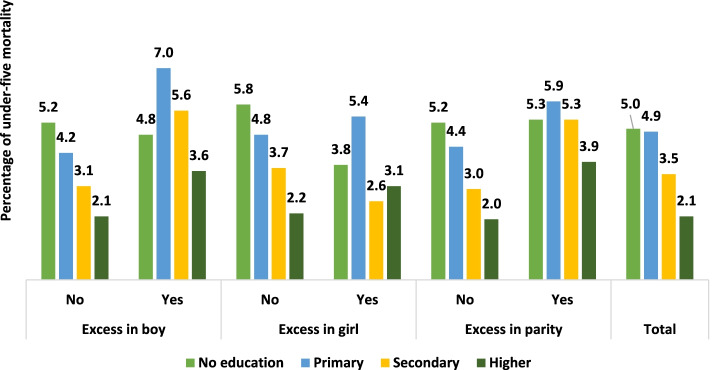
Percentage of under-five mortality rate attributable to undesired children by educational attainment of mother

**Fig. 4:**
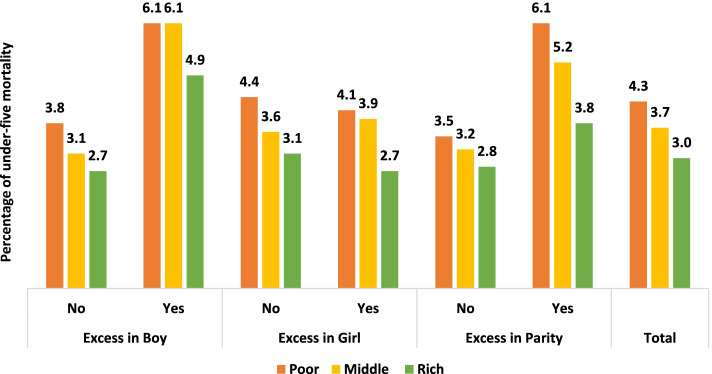
Percentage of under-five mortality rate attributable to undesired children by household wealth index

Childhood morbidity, mortality, and malnutrition varied with the socioeconomic status of the mother. Figure 3 and Fig. 4 show the under-five mortality rates attributable to undesired children by educational attainment and household wealth index. Female education is a significant determinant of fertility and mortality decline. We observed that the under-five mortality rate was the lowest among the higher-educated mothers. Nevertheless, irrespective of educational attainment, the relative contribution of undesired children to under-five mortality was larger for boys, girls, and parity. Similar findings were also observed by the household wealth index, where undesired children experienced higher mortality than desired children.

## Discussion

The efforts had been provided, in this study, to assess the effect of undesired children on childhood mortality, morbidity, and nutritional problems in Bangladesh. The findings showed that the overall undesired children were 19.2%, 21.5%, 3.7%, and 25.4% for boys, girls, both boys and girls, and parity, respectively. Age, education, residence, division, religion, household wealth index, husband's education, access to media, and working status of women were significantly associated with undesired children. The prevalence of under-five mortality was 3.3% among desired children and was almost double among undesired children. Childhood morbidity and malnutrition were also higher and significantly associated with undesired children. However, the postnatal checkup for babies within two days after birth was 17% higher among undesired children.

The findings of this study were both consistent and inconsistent with previous studies [2, 31, 42–45]. There were substantial gender preferences (especially son preferences) in south Asian and African countries [2, 31, 42, 43, 46, 47]. Previous literature from Bangladesh also showed son preference [48, 49], but we observed 2.3% higher unwanted boys than girls. We found that a child undesired to the mother was associated with differential mortality, not by accident or maternal factors (e.g., age). It was consistent with recent literature [2, 45, 50]. The probable explanation would be that if a woman delivers more than she desires, then the children (either gender) may face a lack of proper caring. The reluctance towards unwanted children may sometimes start before birth.

Moreover, previous studies showed that higher childhood mortality was associated with lower socioeconomic status [1, 5, 6]. Female education, a major socioeconomic status, is a significant determinant of fertility and mortality decline. We also found that childhood mortality was higher among lower socioeconomic statuses. Nevertheless, irrespective of educational attainment, the relative contribution of undesired children to under-five mortality was substantial. Similar findings were also observed for the household wealth index. The adjusted model validated the results, similar to existing studies [2, 45].

This study also showed that unintended pregnancy was significantly associated with low birth weight for girls, similar to an existing study [33]. The possible reason was that the mother might be reluctant about the proper diet for undesired pregnancy, resulting in bigger nutritional problems. Unwanted pregnancy may be ended in safe abortion, but abortion is illegal in Bangladesh [14]. As a result, most unwanted pregnancies end in unwanted birth, and unwanted children might suffer from inequitable care.

Undesired girls are significantly associated with an increased likelihood of wasted and being underweight, similar to existing studies [12, 51]. Shaka et al. showed that children from unintended pregnancies were three times more likely to be stunted in southern Ethiopia. Equally, having unwanted or mistimed at conception showed adverse effects on children's growth (especially stunting) when compared with children reported as wanted [50]. The possible reason might be taking extra care of both the mother and child during pregnancy and after the birth of desired children. Similarly, we also found that undesired children were more likely to be infected with fever. However, the postnatal checkup for babies within two days after birth was higher among undesired children as an undesired child might be less healthy than the desired child.

Moreover, though vaccination coverage is very high in Bangladesh, the undesired children had lower coverage regarding Vitamin A supplementation. Marston and Cleland (2003) also showed similar findings for vaccination coverage. Unwanted childbearing has always been associated with increasing poor child health; thus, reducing unwanted childbearing has been essential for improving child health and survival [2, 3, 50].

### Strengths and limitations

This study has some compelling strengths since it was the first study to identify the effect of undesired children on childhood mortality, morbidity, and malnutrition in Bangladesh. We analyzed the nationally representative data, which might increase the acceptability and generalization to similar socioeconomic settings. We utilized multilevel mixed-effects logistic regression analyses for adjusting cluster variations. Despite such strengths, the study has some limitations. Existing studies showed some limitations in measuring unwanted children [2, 31, 37, 38, 40, 52–54], which were also applicable to this study. Unwanted pregnancy might become wanted after birth due to the ethical perspective of mothers, which may underestimate the rate of undesired children [55]. However, we found low undesired children using the direct BDHS question than an indirect approach (using desired and live birth) (supplementary tables). Undesired children might positively be correlated with parity as increasing the number of children increases the rate [2]. We found that among higher parity women, the percentage of undesired children is also higher. The size of the child after birth was mostly self-reported by the mother as less than half of deliveries (49%) were at health facilities [1]. Self-reported data is sometimes vulnerable to recall bias and social desirability bias. Besides, using data from a cross-sectional research design was less than ideal for determining causality. In addition how did a mother deal with an unwanted pregnancy and the child would not be found with a quantitative study. Therefore, a qualitative study and prospective cohort study would bring the best results and explore the relation of the behavioral and psychological factors to pregnancy, births, and child rearing.

## Conclusions

This current study stated that the share of childhood mortality, morbidity, and malnutrition was higher among undesired children. Regarding policies to address childhood mortality and malnutrition in Bangladesh, the findings highlight the concepts of the ideal family size. Limiting unwanted childbearing may reduce some of the differential mortality of undesired children, and unwanted pregnancy may be overcome with the proper utilization of modern contraception. As a result, it will help achieve the SDG of reducing infant and under-five mortality. However, undesired children may be given birth due to desire sex composition. To get the desired sex of children, many women may frequently become pregnant and give birth to undesired sons or daughters. Therefore, a strong message should provide against gender preferences. We can also have a slogan that no unwanted pregnancies are desirable. The government should reemphasize the family planning program to reduce childhood mortality, morbidity, and malnutrition due to unwanted pregnancies and childbirths.

## Supplementary Information

**Additional file 1.** 

## Data Availability

The dataset (BDHS 2017–18) used in this study is publicly available on the DHS website https://dhsprogram.com/methodology/survey/survey-display-536.cfm

## Abbreviations

ANC: Antenatal Care
aOR: Adjusted Odds Ratio
ARI: Acute Respiratory Infection
BDHS: Bangladesh Demographic and Health Survey
ENMR: Early Neonatal Mortality Rate
IMR: Infant Mortality Rate
NMR: Neonatal Mortality Rate
PNMR: Post-Neonatal Mortality Rate
SB: Stillbirths
SDGs: Sustainable Development Goals
U5MR: Under-Five Mortality Rate
