# Supplementary material for: Is there any association between undesired children and health status of under-five children? Analysis of a nationally representative sample from Bangladesh
Source: BMC Pediatr. 2022 Jul 25;22:445. doi: 10.1186/s12887-022-03489-7 (PMC9310505; doi:10.1186/s12887-022-03489-7)
Supplement: Supplementary file 1 — Additional file 1. [file 12887_2022_3489_MOESM1_ESM.docx]

Is there any association between undesired children and health status of under-five children? Analysis of a nationally representative sample from Bangladesh

# **Supplementary materials**

**Table 1** Percentage of wanted children by selected socioeconomic characteristics

| **Background characteristics** | **Wanted last child** | | | **Number** |
| --- | --- | --- | --- | --- |
|  | **wanted then** | **wanted later** | **wanted no more** |  |
| **Sex of last child** |  |  |  |  |
| Male | 78.3 | 14.0 | 7.6 | 2964 |
| Female | 75.2 | 15.6 | 9.2 | 2705 |
| **Year of birth of children** |  |  |  |  |
| 2014 | 63.6 | 26.2 | 10.2 | 431 |
| 2015 | 77.0 | 14.7 | 8.3 | 1671 |
| 2016 | 77.6 | 14.6 | 7.8 | 1765 |
| 2017 | 78.7 | 12.7 | 8.6 | 1728 |
| 2018 | 90.3 | 1.4 | 8.3 | 72 |
| **Current age of women** |  |  |  |  |
| 15-24 | 79.7 | 18.5 | 1.8 | 3034 |
| 20-34 | 75.5 | 11.0 | 13.5 | 2325 |
| 35-49 | 58.9 | 6.5 | 34.6 | 309 |
| **Age of motherhood** |  |  |  |  |
| 11-19 | 80.3 | 18.9 | 0.8 | 1526 |
| 20-34 | 76.6 | 13.7 | 9.7 | 3928 |
| 35-49 | 57.5 | 4.2 | 38.3 | 214 |
| **Place of residence** |  |  |  |  |
| Urban | 75.9 | 16.0 | 8.1 | 1503 |
| Rural | 77.2 | 14.3 | 8.5 | 4165 |
| **Division (Region)** |  |  |  |  |
| Barishal | 73.9 | 15.0 | 11.1 | 314 |
| Chittagong | 80.7 | 11.9 | 7.4 | 1230 |
| Dhaka | 77.6 | 14.4 | 8.0 | 1427 |
| Khulna | 71.4 | 20.1 | 8.5 | 493 |
| Mymensingh | 77.8 | 14.0 | 8.1 | 492 |
| Rajshahi | 78.0 | 15.2 | 6.8 | 651 |
| Rangpur | 72.0 | 17.7 | 10.2 | 586 |
| Sylhet | 75.1 | 14.2 | 10.7 | 478 |
| **Religion** |  |  |  |  |
| Muslim | 76.3 | 15.1 | 8.6 | 5227 |
| Other | 83.7 | 10.9 | 5.4 | 441 |
| **Household wealth quintile** |  |  |  |  |
| Poor | 73.0 | 16.1 | 11.0 | 2391 |
| Middle | 78.7 | 14.0 | 7.2 | 2195 |
| Rich | 81.4 | 13.5 | 5.1 | 1083 |
| **Educational level of women** |  |  |  |  |
| No education | 70.3 | 11.9 | 17.8 | 370 |
| Primary | 71.6 | 16.2 | 12.2 | 1604 |
| Secondary | 79.7 | 13.7 | 6.7 | 2753 |
| Higher | 80.3 | 16.6 | 3.1 | 939 |
| **Husband's education level** |  |  |  |  |
| No education | 71.6 | 13.2 | 15.2 | 783 |
| Primary | 74.1 | 16.0 | 9.9 | 1918 |
| Secondary | 79.3 | 14.0 | 6.7 | 1869 |
| Higher | 80.9 | 15.5 | 3.6 | 1016 |
| **Access to any media for FP** |  |  |  |  |
| No | 75.7 | 15.2 | 9.1 | 4722 |
| Yes | 82.3 | 12.7 | 5.0 | 946 |
| **Current working status of women** |  |  |  |  |
| No | 78.3 | 14.5 | 7.2 | 3562 |
| Yes | 74.4 | 15.3 | 10.3 | 2107 |
| **Women participating in household decision making** |  |  |  |  |
| Low | 76.0 | 15.4 | 8.7 | 208 |
| Moderate | 76.8 | 14.6 | 8.7 | 4687 |
| High | 77.5 | 15.8 | 6.7 | 774 |
| **Wife beating justified** |  |  |  |  |
| No | 77.3 | 14.7 | 8.0 | 4647 |
| Yes | 74.6 | 15.0 | 10.4 | 1020 |
| **Total** | 76.8 | 14.8 | 8.4 | 5667 |

**Table 2** The prevalence of morbidity and mortality among children born in the past five years by the wanted status of children in Bangladesh

| **Childhood Mortality and Morbidity** | **Wanted last child** | | | **Total** | **Number** |
| --- | --- | --- | --- | --- | --- |
|  | **wanted then** | **wanted later** | **wanted no more** |  |  |
| **Indicators of Child Mortality** | | | | |  |
| Early Neonatal Mortality | 3.0 | 1.4 | 1.7 | 2.7 | 5669 |
| Neonatal Mortality | 3.6 | 1.7 | 1.9 | 3.2 | 5668 |
| Post Neonatal Mortality | 1.0 | 1.1 | 0.8 | 1.0 | 5668 |
| Infant Mortality | 4.6 | 2.7 | 2.7 | 4.2 | 5667 |
| Child Mortality | 0.1 | 0.0 | 0.0 | 0.1 | 5667 |
| Under 5 Mortality | 4.7 | 2.7 | 2.9 | 4.3 | 5668 |
| **Indicators of Child Morbidity: Childhood Nutritional Status** | | | | |  |
| Severely Stunted | 9.5 | 10.6 | 10.0 | 9.7 | 5083 |
| Moderately Stunted | 31.0 | 33.5 | 33.0 | 31.5 | 5084 |
| Over-Height for Age | 1.3 | 1.6 | 3.0 | 1.5 | 5084 |
| Severely Underweight | 4.2 | 4.7 | 4.0 | 4.3 | 5241 |
| Moderately Underweight | 19.2 | 21.6 | 23.8 | 20.0 | 5241 |
| Overweight for Age | 0.8 | 0.3 | 0.4 | 0.7 | 5241 |
| Severely Wasted | 1.7 | 1.6 | 3.3 | 1.8 | 5066 |
| Moderately Wasted | 8.5 | 7.1 | 8.9 | 8.3 | 5068 |
| Overweight for Height | 2.5 | 2.1 | 1.4 | 2.3 | 5069 |
| Low Birth Weight (LBW) | 15.2 | 18.7 | 13.4 | 15.5 | 2331 |
| **Indicators of Child Morbidity: Most Common Disease in Childhood** | | | | | |
| Diarrhea in the last two weeks | 6.3 | 7.0 | 4.1 | 6.2 | 5428 |
| Fever in last two weeks | 34.9 | 36.0 | 42.0 | 35.7 | 5427 |
| Cough in the last two weeks | 37.7 | 38.2 | 37.0 | 37.8 | 5427 |
| Short, rapid breaths | 15.5 | 12.3 | 15.2 | 15.0 | 5427 |
| Acute Respiratory Infection | 3.5 | 2.7 | 3.0 | 3.3 | 5427 |
| **Checkups and Treatment** |  |  |  |  |  |
| PNC for baby within 2 days | 42.0 | 42.6 | 55.6 | 43.2 | 4913 |
| Treatment for diarrhea | 49.0 | 42.1 | 31.6 | 46.9 | 337 |
| Treatment for fever/cough | 43.2 | 46.5 | 36.9 | 43.1 | 2123 |
| Vitamin A coverage | 70.3 | 68.4 | 63.2 | 69.4 | 5427 |
